# When a Woman Kills Her Man: Gender and Victim Precipitation in Homicide

**DOI:** 10.1177/0886260519834987

**Published:** 2019-03-08

**Authors:** Karoliina Suonpää, Jukka Savolainen

**Affiliations:** 1 University of Helsinki, Helsinki, Finland; 2 University of Michigan, Ann Arbor, MI, USA

**Keywords:** homicide, victim precipitation, intimate partner, gender, replication

## Abstract

This research revisited the claim that victim precipitation (VP) is especially prevalent in situations where women kill their male intimate partners. Using administrative data from the Finnish Homicide Monitor (*N* =1,494), we created a typology of homicide incidents to examine variation in VP across three factors: the gender of the offender, the gender of the victim, and the intimacy of the victim–offender relationship. The results from regression models demonstrated strong support for the assumption that killings by women of their male intimate partners are more likely to have been victim precipitated than other types of homicide. This homicide type stood out as having the strongest association with each measure of VP included in the analysis. We did not observe statistically significant differences in VP among other homicide types. For example, we did not observe gender differences in VP in homicides that did not involve intimate partners. This pattern of results contradicts prior evidence suggesting that VP is a general feature of female-perpetrated killings, independent of the gender of the victim and the intimacy of the victim–offender relationship. As such, the present study underscores the importance of replication in studies of interpersonal violence. Theoretically, the results support the gender–partner interaction hypothesis over gender differences hypothesis of VP.

Much of interpersonal violence stems from the escalation of bilateral disputes (e.g., Felson, 1993/2017; Griffiths, Yule, & Gartner, 2011; Luckenbill & Doyle, 1989). For example, a grievance in traffic may elicit an angry reaction, with the potential for provoking retaliation. Depending on the environmental circumstances and the individual characteristics of the parties involved, the ensuing “character contest” may escalate into a violent episode of road rage. Sometimes, it is difficult to determine who started the fight as both parties think, with reasonable validity, that it was the other person. In these situations, the term “victim” is usually applied to the person who endured the greatest physical harm, even if that person instigated the incident. If the outcome is homicide, the victim is the person who was killed.

The focus of the present study is on victim-precipitated homicide. As defined by Wolfgang (1967), victim-precipitated homicide refers to a lethal act in which “the role of the victim is characterized by his having been the first in the homicide drama to use physical force directed against his subsequent slayer” (p. 73). In contemporary research literature, victim is understood to have precipitated the incident if (s)he was the first to have resorted to physical violence (e.g., Felson & Messner, 1998; Jurik & Winn, 1990; Muftić, Bouffard, & Bouffard, 2007; Muftić & Hunt, 2012). Wolfgang argued that victim precipitation (VP) is a common feature of *intimate partner homicides* (IPHs), especially in situations involving a female offender and a male victim (Wolfgang, 1957, 1967).

There is considerable support in the literature for the assumption that homicides involving female offenders are more likely to have been victim precipitated. Jurik and Winn (1990) found that 67% of male perpetrators could be described as the ones who had “turned the confrontation into a physical attack,” compared with 36% of female killers. Yourstone, Lindholm, and Kristiansson (2008) reported that 51% of female offenders but only 14% of male offenders had been physically abused by the victim prior to the lethal incident. A study of IPH found that self-defense played a role in 36% of the cases involving a female killer, compared with only 1% of male-perpetrated homicides of the female partner (Weizmann-Henelius et al., 2012). Finally, Jordan, Clark, Pritchard, and Charnigo (2012) found that as many as 66% of female but only 2% of male perpetrators of lethal or life-threatening violence toward their partners reported prior experience with physical, sexual, or psychological victimization as adults—although not necessarily at the hands of their intimate partner.

Battered wife syndrome (Walker, 1979, 2017), or some less extreme version of it, is a frequent explanation for why VP is presumed to be particularly prevalent in situations where women kill their male partner (Daly & Wilson, 1988; Dobash, Dobash, Wilson, & Daly, 1992; Serran & Firestone, 2004; Wilson & Daly, 1998). The assumption is that killings of this variety are frequently motivated by either acute self-defense or the desire to end an abusive relationship, that is, to escape circumstances sometimes described as “intimate terrorism” (Johnson & Ferraro, 2000). Women may be motivated to kill an abusive partner if they are in danger of being killed or if they believe their children are in mortal danger (Johnson, 1995; Johnson & Ferraro, 2000). Prior research confirms the expectation that self-defense is an important context of female-perpetrated intimate partner homicide (F-M IPH); yet, it is seldom a motive for IPH committed by men against their female partners (Felson & Messner, 1998; Weizmann-Henelius et al., 2012).

Because most studies of IPH do not compare these outcomes with homicides that did not involve intimate partners, they cannot rule out the possibility that VP is equally central in killings by women of men (and women), *regardless of the intimacy* of the victim–offender relationship. As noted by Felson and Messner (1998), to demonstrate that VP is a special feature of F-M IPH of men, one must demonstrate a three-way interaction between (a) the gender of the offender (female), (b) the gender of the victim (male), and (c) the nature of relationship between the victim and the offender (intimate partners). In their research, Felson and Messner (1998) contrasted this *gender–partner interaction hypothesis* with two alternative hypotheses, both of which offer more parsimonious accounts of the patterns typically observed in the literature.

First, the *gender interaction hypothesis* assumes that, regardless of the intimacy of the relationship, homicides involving female offenders and male victims are more likely to be victim precipitated. In other words, according to this hypothesis, intersexual (as opposed to intrasexual) killings by women are more likely to be victim precipitated because men in general, not just male partners, are more likely to instigate physically violent episodes with women. In situations where interpersonal dispute escalates into lethal violence, women are less likely than men to be the first to engage in life-threatening behavior. Thus, when a woman kills a man, the event is more likely to have been motivated by self-defense as compared with incidents where a woman kills another woman.

Second, the *gender differences hypothesis* suggests that, regardless of the gender of the victim, all female-perpetrated homicides are more likely to involve VP because women are generally less violent than men and, thus, require more provocation to resort to lethal acts of violence. Symmetrically, it follows from this assumption that, regardless of the gender of the offender, homicides involving male victims are more likely to be victim precipitated: “These effects should occur whether the offender is a man or woman or whether the offender is his or her partner” (Felson & Messner, 1998, p. 409).

The analyses performed by Felson and Messner (1998) failed to sustain the *gender–partner interaction* model, but found support for the *gender differences* hypothesis. In other words, their results suggest that “the fact that men tend to be more violent than females” (p. 405) is sufficient to account for the observed patterns. To our knowledge, this is the only prior study of VP in homicide that has attempted to disentangle the effects of gender and intimacy. Notably, this research found no support for the commonly held assumption that VP is especially prevalent in situations where women kill their male intimate partners.

Because evidence from a single study is not sufficient to establish a scientific fact (Lakatos, 1970), we think it is important to revisit this topic. As described below, we pursued the same question as Felson and Messner (1998), but used data and methods that are different from the original study. As such, the present study can be understood as a type of replication known as *generalization*, the purpose of which is to investigate whether “similar findings may be observed consistently across methods and settings” (Freese & Peterson, 2017, p. 152). Revisiting Felson and Messner is all the more important in light of the fact that the evidence presented in that study contradicts a major assumption in the literature on intimate partner violence.

## Current Study

### Hypothesis

This research is focused on VP in homicide. Following Felson and Messner (1998), the goal is to disentangle the independent and joint effects of the gender of the *victim*, the gender of the *offender*, and the victim–offender *relationship* on IPH. The hypothesis we pursue predicts that F-M IPHs are more likely to be victim precipitated than other combinations of gender and relationship intimacy. As noted, Felson and Messner (1998) did not find support for this hypothesis.

### Data and Analytic Strategy

The data come from the Finnish Homicide Monitor (FHM), which is designed to track all homicide incidents that have taken place in Finland since July 1, 2002. Recorded by the chief investigating officer of the criminal case, the data elements provide information about the sociodemographic characteristics of the victims and the offenders, the nature of their relationship, their criminal histories, their behavior at the crime scene, and the motive as determined by the investigating officer. The FHM is maintained jointly by the Finnish National Police, Institute of Criminology and Legal Policy at the University of Helsinki, and the Police College of Finland (for more information of FHM, see, for example, Kivivuori & Lehti, 2012).

The data for the current study were retrieved from the FHM on January 15, 2019. At that time, the database covered all homicide cases from July 2002 through December 2017 (*N* = 1,673 victims). The data file is victim based. If the incident involved multiple offenders, the “offender” refers to the principal offender. Given the research question, we omitted cases featuring victims (*n* = 116) and offenders (*n* = 23) below the age of 18. We also excluded cases that did not include any information about the key variables of interest, that is, the victim–offender relationship and the presence of VP (*n* = 58). Finally, due to the small number of cases involving same-sex couples (*n* = 8), the analysis is limited to heterosexual couples. The resulting analysis sample consists of 1,494 homicide cases that meet the inclusion criteria.

#### Measures

##### VP

The FHM database features four indicators that can be used to measure VP. The first such item indicates whether the offender used lethal violence “at least partly for self-defense.” The second item indicates whether the motive of the offender was to “end repeated acts of violence or abuse against oneself.” The third measure of VP indicates whether the homicide victim had “a history of violence against the offender prior to the lethal incident.” Finally, the fourth VP measure indicates whether the victim “had threatened to use violence against the offender in the past.”

Note that the first two indicators concern motives directly related to the killing (i.e., the instant case), whereas the last two items are about past behavior. In the latter situation, VP is inferred on the basis of past behavior. Note also that the first indicator of VP (self-defense) is focused strictly on the immediate situation in which the homicide occurred, whereas the second indicator (ending violence/abuse) implies a persistent pattern of behavior by the victim against the offender. These codes are not mutually exclusive; multiple indicators of VP may apply to any actual homicide incident.

Using information from these four items, we created two “global” measures of VP. The first one is a simple dichotomy indicating the presence of any one of those incident characteristics (1 = yes, 0 = no).^1^ The second measure of VP is a count variable indicating how many VP items were observed in the homicide incident. The values of this variable range from 0 to 4. In addition, we report results from models featuring each individual VP item as the dependent variable. Each one of those is measured as a dichotomy (1 = yes, 0 = no).

##### Gender and intimacy

As described in Table 1, we created six homicide types by combining information from three binary incident characteristics: victim’s gender, offender’s gender, and the victim–offender relationship (intimate partner vs. other). The “intimate partner” classification includes those who were living in a domestic union, either married or cohabiting, romantic partners (boyfriend/girlfriend), as well as former partners (e.g., ex-wife).^2^

**Table 1.** Six Homicide Types Representing the Combinations of Three Dichotomous Incident Characteristics: Offender’s Gender, Victim’s Gender, and the Victim–Offender Relationship.

Homicide Type	Offender’s Gender	Victim’s Gender	Victim–Offender Relationship	%	*N*
F-M IPH	Female	Male	Intimate partner	4.6	69
F-M	Female	Male	Other	3.3	50
F-F	Female	Female	Other	1.4	21
M-F IPH	Male	Female	Intimate partner	19.0	284
M-F	Male	Female	Other	9.4	141
M-M	Male	Male	Other	62.2	929
Total				100	1,494

*Note*. F-M IPH = female-perpetrated intimate partner homicide; M-F IPH = male-perpetrated intimate partner homicide; F-F = all-female killing; M-M = all-male killing; F-M = female–male not IPH; M-F = male–female not IPH.

##### Control variables

Given concerns about statistical power, we limited the number of control variables to the age of the victim and the offender.^3^

#### Analytic approach

We estimated logistic regression equations for models featuring a binary outcome. The count measure of VP was examined using negative binomial regression, which is a Poisson-based model for overdispersed outcomes. In departure from Felson and Messner (1998), we did *not* construct multiplicative interaction terms. Instead, we treated the six homicide categories as a set of dummy variables and estimated their main effects on VP. Using F-M IPH as the reference category, the resulting coefficients indicate the degree of VP present in the other homicide categories relative to F-M IPH—our focal category of interest. The hypothesis is that, compared with F-M IPH, the other homicide categories will be associated with negative coefficients. To assist in the interpretation of these nonlinear estimates, we report the average marginal effects (AMEs), which measure the change in the probability of VP for each homicide category vis-à-vis the reference category at observed values of the other covariates (Mood, 2010; William, 2012).

We prefer this modeling approach because it is more transparent and less problematic statistically than estimating multiplicative interaction terms between variables. First, estimating a three-way interaction term across three dichotomous variables yields eight categories (2 × 2 × 2 = 8), all of which remain opaque when captured by a single regression coefficient. It would be impossible for the reader to see which combinations are responsible for the observed effect. Moreover, because there are no same-sex couples in the analysis, including a three-way interaction term between offender’s gender, victim’s gender, and the intimacy of the relationship would have created multicollinearity between variables. Note that Felson and Messner (1998) reported similar problems in their study. Second, although the use of multiplicative interaction terms is widespread in social sciences, there are important, but frequently ignored, issues with the interpretation of such effects in nonlinear models (Ai & Norton, 2003). In logistic regression, the statistical significance of the interaction term cannot be tested with a simple *t* test, and even the sign of the coefficient associated with the same interaction effect can be different from what it would be, had it been estimated using linear regression (Ai & Norton, 2003; Ganzach, Saporta, & Weber, 2000; Karaca-Mandic, Norton, & Dowd, 2012). Our approach overcomes these methodological challenges, yielding estimates that are easy to interpret.

## Results

Descriptive statistics for the analytic variables are presented in Tables 1 and 2. Table 1 reports the frequency and distribution of homicide incidents across the six homicide types. Not surprisingly, male-on-male homicides dominate the caseload (62.2%). The second most common type is IPH in which a man kills his female partner (19.0%). There are very few all-female killings (F-F) in this database (*n* = 21, 1.4%). Turning to Table 2, approximately one third (32.8%) of homicides included evidence of some VP. The count measure of VP shows that most (68.5%) homicides did not feature any VP, but as many as 44 homicides were coded as having evidence of each of the four VP indicators. Turning to the individual items of VP, we observe that 12.0% of the homicides were motivated by self-defense, and as much as 13.2% were motivated by the desire to end an ongoing situation of violence and abuse. Evidence of prior violence and threats toward the offender was recorded in approximately one out of five homicides. The average age of the victims in this sample was 8 years older than the offenders’ (46 years vs. 38 years).

**Table 2.** Descriptive Statistics.

Measures	%	Range	*M* (*SD*)	Valid *N*
I. VP				
A. Pooled measures of VP^a^				
Any VP	332.8	0-1		1,494
VP count	1100	0-4		1,155
None	668.5			791
One	113.9			160
Two	88.7			101
Three	55.1			59
Four (all)	33.8			44
B. Individual VP items				
Self-defense	112.0	0-1		1,439
Ending abuse	113.2	0-1		1,436
Victim’s prior violence	221.2	0-1		1,325
Victim’s prior threats	119.9	0-1		1,252
II. Control variables				
Offender age		18-90	38.4 (14.0)	1,494
Victim age		18-91	45.5 (15.1)	1,494

*Note.* VP = victim precipitation.

a These measures combine information from the four VP items listed under heading B.

We estimated five binary logistic regression equations and one negative binomial regression model. In what follows, we report the key results in graphs that plot AMEs using 95% confidence intervals (CIs). The tables reporting the regression coefficients are provided in the Online Appendix. In each model, F-M IPH is treated as the reference category. Thus, the theoretical expectation is to observe *negative effects* for each homicide type displayed in the graphs. The results were adjusted for the age of the victim and the offender.

**Figure 1.**
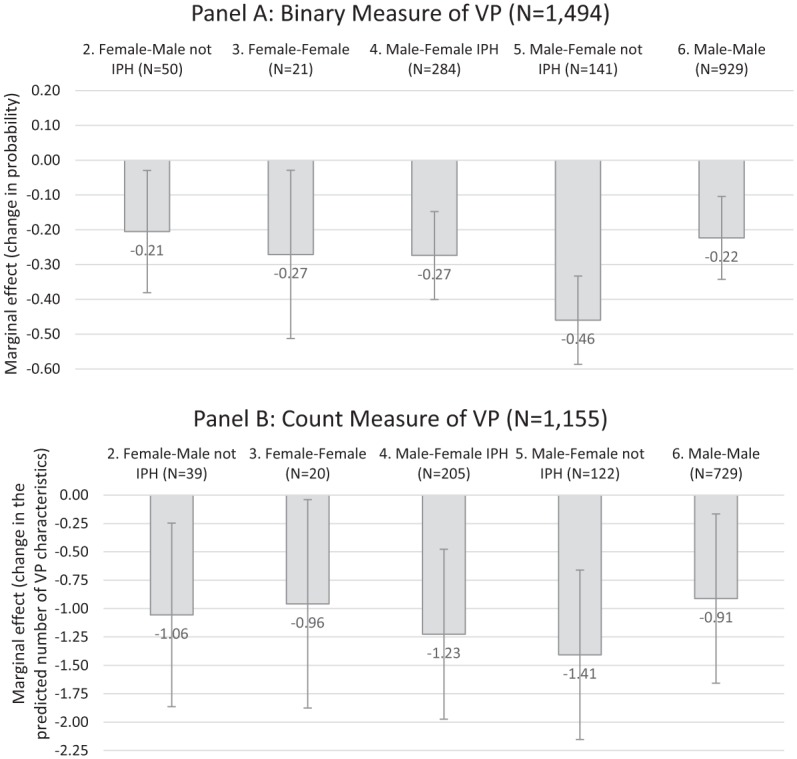
The association between homicide type and VP. *Note.* Average marginal effects from logistic (Panel A) and negative binomial (Panel B) regression models. In each model, the reference category is female–male IPH. IPH = intimate partner homicide; VP = victim precipitation.

Panel A of Figure 1 presents the AME estimates of the relationship between homicide type and the dichotomous indicator of *any* VP. In support for the hypothesis, each estimate is negative and statistically significant, as indicated by the fact that the CIs stay below zero. This pattern implies that the reference category (F-M IPH) is more likely to involve victim-precipitated killings than any other homicide type. For instance, when women kill males who are not their intimate partners, there is a 21 percentage point lower probability of VP (AME = −0.21, 95% CI = [−0.38, −0.03]) than when women kill their intimate partners.

This result supports the *gender–partner interaction* hypothesis predicting more VP within the F-M IPH category. The results contradict the *gender interaction* hypothesis because we observe VP to be more prevalent in situations where women kill their male intimate partners than when they kill other men. In addition, we find no support for the gender differences hypothesis because there is less VP also in all-female (F-F) killings than in the F-M IPH (reference) category. The gender differences hypotheses predicts no differences in VP among varieties of female-perpetrated homicide.

Panel B of Figure 1 presents results from the analysis featuring the *count measure* of VP. The overall patterns are similar to the ones observed above: All AMEs are negative and statistically significant at the .05 level. To be sure, the F-F category is close to the critical level (*p* = .041), but note that this category features a small number of valid cases (*n* = 20) in this model.

**Figure 2.**
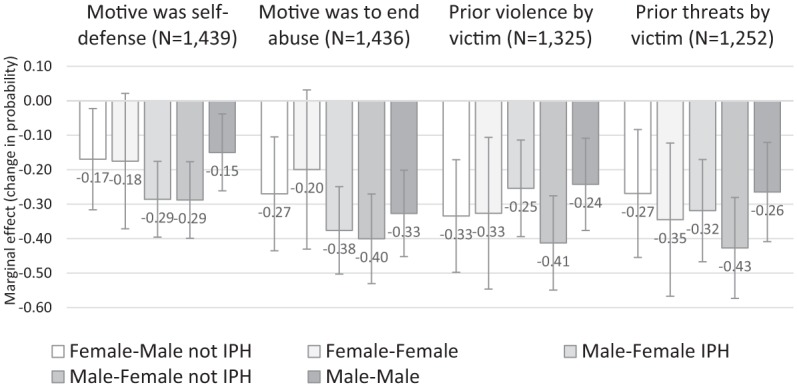
The association between homicide type and four dichotomous indicators of victim precipitation. *Note*. Average marginal effects from logistic regression models. In each model, the reference category is female–male IPH. IPH = intimate partner homicide.

Figure 2 presents the final set of results. This graph presents information from four different logistic regression models, each of which features a single VP indicator as the dependent variable: *self-defense, ending abuse, prior violence*, and *prior threats*. (The previous analyses were based on global measures of VP, which pooled information from each of these four indicators.) Consistent with the previously reported findings, all the estimates displayed in Figure 2 are negative. The CI associated with F-F category overlaps with the reference category in models featuring *self-defense* and *ending abuse* as indicators of VP. As noted above, this homicide category includes few cases resulting in wide CIs. In the models predicting the presence of prior violence or abuse by the victim, all the estimates are different from F-M IPH (the reference category) by a statistically significant margin (*p* < .05).

The results from the main analyses, reported above, are based on data that included homicides featuring multiple victims and/or offenders. Because these kinds of incidents comprise a nontrivial minority of cases (*n* = 353, 23.6%), we repeated the analyses using data limited to single victim/offender incidents (*n* = 1,141). The results from this sensitivity check reproduced the patterns observed in the main analysis with one exception: In the model featuring the binary measure of *any VP*, the CI for the F-M category overlapped with the reference category (AME = −0.15, 95% CI = [−0.35, 0.05]). In other words, female-perpetrated killings of nonintimate males did not differ by a statistically significant margin from the F-M IPH category. However, this result was limited to only one measure of VP. The difference was statistically significant in all the other models, as it was in this model using the complete data file. Finally, with respect to the F-F category, using the reduced sample also “improved” the results somewhat: In the model featuring the self-defense item as the measure of VP, the CI for the coefficient associated with F-F did not overlap with the reference category, unlike in the equivalent model reported in the main analysis (Figure 2).

## Discussion

It is frequently suggested in the criminological literature that VP plays a particular role in situations where women kill their male partners. Originally formulated by Wolfgang (1967), this assumption looms large in more general discussions of gender and violence (Dobash et al., 1992; Johnson, 2006). Despite its theoretical appeal, most prior studies of IPH have failed to demonstrate *critical support* for the hypothesis because they have ignored alternative explanations, such as the gender differences hypothesis, which argues that women in general, regardless of the characteristics of their adversaries, need more provocation than men to engage in serious violence.

Following the analytic approach developed by Felson and Messner (1998), the present study considered the impact of three factors on VP: the gender of the offender, the gender of the victim, and the intimacy of the victim–offender relationship. We created a typology of homicide incidents to examine the unique and joint effects of each factor. The results largely confirmed the expectation that VP motivates female killings of their male partners more than other configurations of gender and victim–offender relationship.

The findings were similar across six operationalizations of VP. We observed some instability in the statistical significance of the results comparing F-M IPH against F-F killings. However, this was likely due to the small number of incidents in the F-F category as the magnitude of the coefficients associated with F-F were strongly negative across each model specification. We did not observe any statistically significant differences among other homicide types. Crucially, female-perpetrated killings that *did not* involve intimate partners were no more likely to have been victim precipitated than homicides committed by males. These results contradict Felson and Messner’s (1998) prior study, which favored the gender differences hypothesis.

The discrepancy between the two studies is noteworthy because the present study is the first replication of the only prior attempt to disentangle the effects of gender and intimacy on VP. Our research was based on a different data source and used a different methodological approach than what was used in the original study by Felson and Messner (1998). We cannot determine whether the differences in the results are related to the data source, the method, or both. We have argued that, in light of the current literature on nonlinear modeling, and in the situations with small or nonexistent number of some subtypes—such as all-female homicides or those involving same-sex intimate partners—using a transparent typology of homicide incidents is recommended over the multiplicative estimation strategy embraced by Felson and Messner (1998).

It would be helpful to know what the results were if the method used in the present study was applied to the data used in the original research. In light of prior literature, we would expect the patterns observed in the Finnish data to emerge even stronger in the United States. This assumption is based on evidence suggesting that, due to differences in gender stratification, “Finnish women are less likely that their U.S. counterparts to be subjected to abusive intimate relationships, and when they are they are better able to escape these relationships without ‘killing their way out’” (Messner & Savolainen, 2001, p. 53). We would like to see this hypothesis examined in future research.

## Limitations

Finland is a small country where homicide is a rare population event. Thus, although we had access to all homicide incidents from close to 16 years, some of the incident categories used in the analysis had only a small number of cases. Most notably, there were too few same-sex IPHs in the data to permit meaningful analysis. The literature on intimate partner violence would benefit from more research on this understudied population. In addition, there were only 21 incidents of all-female killings (F-F), which resulted in wide CIs for estimates pertaining to this homicide category. One way to increase the sample size in future research is to merge data from Finland with comparable data from other nations (Granath et al., 2011; Liem et al., 2013).

The indicators used to assess VP are based on the judgment of the police officers assigned to the case. Although these judgments are constrained by guidelines and informed by training and experience, it is nevertheless conceivable that they are biased by gender. For example, all else equal, the investigating officer may be more likely to see evidence of self-defense if the victim was a male and the offender was a female. This methodological challenge is difficult to overcome with administrative data, and there are obvious limitations to using surveys in homicide research, as “dead men tell no tales.” We can imagine an experimental study using vignettes to test the hypothesis that the gender of the offender/victim affects the judgments of the investigating officers. In the absence of such evidence, we simply acknowledge the possibility of gender bias in the measures of VP.

## Conclusion

Although replication research has always been an accepted feature of social science, an emerging consensus suggests that this line of inquiry has been neglected to the detriment of theoretical progress (Freese & Peterson, 2017; McNeeley & Warner, 2015; Savolainen & VanEseltine, 2018). A recent review of replication research in criminology found that only 0.45% of articles in the *Web of Science* database qualified as replications (Pridemore, Makel, & Plucker, 2018). The current study reduces this deficit by replicating the only prior study adjudicating between three perspectives on VP in homicide. Contrary to the original study, we found considerable support for the widely held belief that the intimacy of the victim–offender relationship matters to VP, along with the gender of the offender and the victim. Note that because we used different data and methods than Felson and Messner (1998), our findings present no challenge to the integrity of their research. To the contrary, while waiting for results from additional replications, their original contribution should be recognized for its theoretical clarity, which helped us, and should help others, to advance etiological research on interpersonal violence.

## Supplemental Material

Appendix – Supplemental material for When a Woman Kills Her Man: Gender and Victim Precipitation in Homicide Click here for additional data file. Supplemental material, Appendix for When a Woman Kills Her Man: Gender and Victim Precipitation in Homicide by Karoliina Suonpää and Jukka Savolainen in Journal of Interpersonal Violence

## Notes

1. The amount of missingness varied across the four measures of victim precipitation (4%-16%). The binary variable was coded in an inclusive manner: Incidents with valid data on at least one of the items were included in the analysis. The count variable, instead, was based on a stricter coding decision: Only the cases with valid data on all of the four measures were included in the analysis.

2. All these designations are based on the assessment by the investigating officer. In the case of (previous) marriage and (previous) cohabitation, the relevant information can be determined from administrative records available to the police. The situation is more subjective for romantic partners. However, the coding manual used by the police instructs them to focus on long-term relationships and to exclude casual sexual partners or short-lived affairs.

3. In analyses not reported here, but available from the authors, we also included immigrant status and socioeconomic status as additional covariates. We omitted those from the final results, as they had no substantive impact on the observed patterns.
