# Supplementary material for: When a Woman Kills Her Man: Gender and Victim Precipitation in Homicide
Source: J Interpers Violence. 2019 Mar 8;34(11):2398–413. doi: 10.1177/0886260519834987 (PMC6537166; doi:10.1177/0886260519834987)
Supplement: Appendix – Supplemental material for When a Woman Kills Her Man: Gender and Victim Precipitation in Homicide [file Appendix.pdf]

Appendix 1: Association between homicide type and the dichotomous indicator of victim precipitation (VP), logistic regression.

	Model 1: Binary Measure of VP (N=1,494)			
	Beta	95 % C.I.		OR
**Homicide type**[a]		Lower	Upper	
F-M not IPH (N=50)	-0.86*	-1.61	-0.10	0.42*
F-F (N=21)	-1.16*	-2.30	-0.02	0.31*
M-F IPH (N=284)	-1.17*	-1.72	-0.63	0.31*
M-F not IPH (N=141)	-2.40*	-3.13	-1.68	0.09*
M-M (N=929)	-0.94*	-1.44	-0.44	0.39*
**Controls**				
Victim's age	-0.02*	-0.03	-0.01	0.98*
Offender's age	0.01	0.00	0.02	1.01
**Constant**	1.13*	0.50	1.76	3.10*

χ^2=88.95, *p<0.05

[a]Reference category F-M IPH (n=69)

Appendix 2: Association between homicide type and the number of measures of victim precipitation (VP), negative binomial model.

	Model 2: Count Measure of VP (N=1,155)			
	Beta	95 % C.I.		IRR
**Homicide type**[a]		Lower	Upper	
F-M not IPH (N=39)	-1.07*	-1.81	-0.32	0.34*
F-F (N=20)	-0.90	-1.86	0.05	0.40
M-F IPH (N=205)	-1.43*	-1.98	-0.89	0.24*
M-F not IPH (N=122)	-2.07*	-2.74	-1.40	0.13*
M-M (N=729)	-0.84*	-1.31	-0.36	0.43*
**Controls**				
Victim's age	-0.02*	-0.03	-0.01	0.98*
Offender's age	0.01	0.00	0.02	1.01
**Constant**	1.19*	0.60	1.77	3.28*

χ^2=84.81, *p<0.05

[a]Reference category F-M IPH (n=40)

Appendix 3: Association between homicide type and the dichotomous indicator of four different measures of victim precipitation, logistic regression.

	Model 3A: Motive was self-defense (N=1,439)			
	Beta	95 % C.I.		OR
**Homicide type**[a]		Lower	Upper	
F-M not IPH (N=46)	-1.06*	-2.08	-0.04	0.35*
F-F (N=21)	-1.11	-2.69	0.47	0.33
M-F IPH (N=274)	-3.24*	-4.28	-2.21	0.04*
M-F not IPH (N=140)	-3.38*	-4.87	-1.88	0.03*
M-M (N=893)	-0.90*	-1.46	-0.33	0.41*
**Controls**				
Victim's age	-0.03*	-0.04	-0.01	0.97*
Offender's age	0.01	-0.01	0.02	1.01
**Constant**	0.08	-0.74	0.90	1.08

χ^2=106.08, *p<0.05

[a]Reference category F-M IPH (n=65)

	Model 3B: Motive was to end abuse (N=1,436)			
	Beta	95 % C.I.		OR
**Homicide type**[a]		Lower	Upper	
F-M not IPH (N=49)	-1.30*	-2.19	-0.42	0.27*
F-F (N=21)	-0.89	-2.03	0.25	0.41
M-F IPH (N=270)	-2.27*	-2.92	-1.61	0.10*
M-F not IPH (N=131)	-2.65*	-3.56	-1.73	0.07*
M-M (N=903)	-1.74*	-2.28	-1.20	0.18*
**Controls**				
Victim's age	-0.01	-0.02	0.00	0.99
Offender's age	0.01	0.00	0.03	1.01
**Constant**	-0.11	-0.87	0.64	0.89

χ^2=62.44, *p<0.05

[a]Reference category F-M IPH (n=62)

	Model 3C: Prior violence by victim (N=1,325)			
	Beta	95 % C.I.		OR
**Homicide type**[a]		Lower	Upper	
F-M not IPH (N=45)	-1.79*	-2.82	-0.77	0.17*
F-F (N=20)	-1.73*	-3.31	-0.14	0.18*
M-F IPH (N=241)	-1.21*	-1.84	-0.58	0.30*
M-F not IPH (N=134)	-2.78*	-3.73	-1.84	0.06*
M-M (N=832)	-1.14*	-1.72	-0.57	0.32*
**Controls**				
Victim's age	-0.03*	-0.04	-0.02	0.97*
Offender's age	0.00	-0.01	0.02	1.00
**Constant**	1.00*	0.25	1.74	2.71*

χ^2=78.65, *p<0.05

[a]Reference category F-M IPH (n=53)

	Model 3D: Prior threats by victim (N=1,252)			
	Beta	95 % C.I.		OR
**Homicide type**[a]		Lower	Upper	
F-M not IPH (N=43)	-1.29*	-2.24	-0.34	0.28*
F-F (N=21)	-1.84*	-3.44	-0.24	0.16*
M-F IPH (N=230)	-1.62*	-2.32	-0.93	0.20*
M-F not IPH (N=134)	-2.86*	-3.83	-1.89	0.06*
M-M (N=779)	-1.26*	-1.88	-0.64	0.28*
**Controls**				
Victim's age	-0.03*	-0.04	-0.02	0.97*
Offender's age	0.00	-0.01	0.02	1.00
**Constant**	1.18*	0.38	1.98	3.26*

χ^2=80.54, *p<0.05

[a]Reference category F-M IPH (n=45)
